# Facile Preparation of Polyacrylonitrile-Based Activated Carbon Fiber Felts for Effective Adsorption of Dipropyl Sulfide

**DOI:** 10.3390/polym16020252

**Published:** 2024-01-16

**Authors:** Tianhao Zhang, Yafang He, Shiqi Hu, Jianlong Ge, Tianye Chen, Haoru Shan, Tao Ji, Decheng Yu, Qixia Liu

**Affiliations:** 1 National & Local Joint Engineering Research Center of Technical Fiber Composites for Safety and Protection, School of Textile and Clothing, Nantong University, Nantong 226019, Chinahrshan@ntu.edu.cn (H.S.); ji.t@ntu.edu.cn (T.J.); 2 Jiangsu Sutong Carbon Fiber Co., Ltd., Nantong 226005, China

**Keywords:** polyacrylonitrile, liquid-phase pre-oxidation, activated carbon fibers, chemical warfare agent simulant adsorption, dipropyl sulfide

## Abstract

Activated carbon fibers (ACFs) derived from various polymeric fibers with the characteristics of a high specific surface area, developed pore structure, and good flexibility are promising for the new generation of chemical protection clothing. In this paper, a polyacrylonitrile-based ACF felt was prepared via the process of liquid phase pre-oxidation, along with a one-step carbonization and chemical activation method. The obtained ACF felt exhibited a large specific surface area of 2219.48 m^2^/g and pore volume of 1.168 cm^3^/g, as well as abundant polar groups on the surface. Owing to the developed pore structure and elaborated surface chemical property, the ACF felt possessed an intriguing adsorption performance for a chemical warfare agent simulant dipropyl sulfide (DPS), with the highest adsorption capacity being 202.38 mg/g. The effects of the initial concentration of DPS and temperature on the adsorption performance of ACF felt were investigated. Meanwhile, a plausible adsorption mechanism was proposed based on the kinetic analysis and fitting of different adsorption isotherm models. The results demonstrated that the adsorption process of DPS onto ACF felt could be well fitted with a pseudo-second-order equation, indicating a synergistic effect of chemical adsorption and physical adsorption. We anticipate that this work could be helpful to the design and development of advanced ACF felts for the application of breathable chemical protection clothing.

## 1. Introduction

In recent years, the frequent occurrence of hazardous chemical leakage accidents and the potential terrorist attacks involving chemical warfare agents pose a serious threat to human health and public security. If these incidents occur without protection, the chemicals can enter the human body through the skin or respiratory tract, causing immeasurable harm to the individuals at the scene [1,2,3]. Chemical protective clothing is a type of personal protective equipment designed to withstand the damage caused by harmful chemicals to the human body. Therefore, the application and development of chemical protective clothing is of great importance. Among various types of chemical protective clothing, breathable chemical protective clothing holds promise as it not only can provide excellent protection, but also allows the passage of air and moisture, significantly improving the physiological comfort of the wearer; thus long-term wear does and will not induce feelings of suffocation or stuffiness, greatly enhancing the mobility of the personnel wearing the garments [4]. As a result, breathable chemical protective clothing is playing an increasingly vital role in the field of individual biochemical protection and has gradually become a research focus [5].

In general, the core component of breathable chemical protective clothing is the adsorption layer. Therefore, the protective performance of these garments highly depends on the performances of the utilized adsorbents [6]. To date, numerous advanced adsorbents with different chemical components and porous structures have been developed for the adsorption of chemical warfare agents, such as porous organic nanomaterials [7], carbon nanotubes [8], metal oxide nanoparticles [9], metal–organic framework materials [10], etc., all of which exhibit well-developed pore structures and a high specific surface area, which are beneficial for the adsorption of chemical warfare agents. However, in addition to these adsorption characteristics, the adsorption layer materials for chemical protective clothing also need to have good chemical stability, good mechanical properties, and processability, as well as relatively low production costs [4]. As a result, activated carbon materials, with the advantages of a high adsorption performance, large adsorption capacity, fast adsorption speed, and low cost, have been widely used for the development of chemical protective clothing. For example, the chemical protective material developed by the German BLÜCHER company based on the “Saratoga spherical activated carbon patent technology”, is currently recognized as one of the most advanced breathable chemical protective clothing materials in the world [11].

Recently, Sharma et al. [12] developed a chemical protective suit with spherical activated carbon as the inner adsorption material, and the results showed that the effective protection time of this protective suit against sulfur mustard could be more than 24 h, considering that sulfur mustard is one of the most dangerous chemical warfare agents due to its ease of manufacture and high destructiveness and is often used to evaluate the performance of chemical protective materials. However, due to the high toxicity and strict control of sulfur mustard, some chemicals such as dipropyl sulfide (DPS), with a similar chemical structure and lower toxicity, have also been used as a simulator of a chemical warfare agent for research in the lab [13,14]. For example, YANG et al. [15] used nano-metal modified spherical activated carbon and used it as an adsorbent for the adsorption of DPS; the result showed that the equilibrium adsorption capacity of modified SAC towards DPS increased by 13.41% compared with that of pristine SAC, and the maximum adsorption capacity could be up to 34.34 mg/g. However, the spherical activated carbon used in protective clothing still suffers from some problems, such as its relatively low adsorption speed, its ease of falling off the cloth, and the insufficient utilization of pore structures due to the coverage of adhesive.

Activated carbon fibers (ACFs) are a newly generated kind of carbonaceous adsorbents with high specific surface areas and developed pore structures. They have the advantages of a large adsorption capacity, fast adsorption speed, convenient processing, and they are ease to compound with other fabrics, showing great potential in the application of breathable chemical protective clothing [6,16]. In general, ACFs are usually made from various polymeric fibers via pre-oxidation, carbonization, activation, and further modification steps, and different fiber precursors and preparation processes will affect the performance of the obtained ACFs. Among various precursors, polyacrylonitrile (PAN) fiber is one of the most used raw materials for the preparation of ACFs. Compared to the other precursors, the PAN based ACFs (PAN-ACFs) possess better mechanical properties and good surface chemical activity with more nitrogen/oxygen functional groups [17]. As for the preparing of PAN-ACFs, the pre-oxidation process plays a critical role in enhancing the thermal stability of PAN fibers. This process ensures that the fibers can withstand high-temperature carbonization activation without melting or combusting to maintain a fibrous structure [18]. The gas-phase pre-oxidation method is the most commonly used approach for producing PAN-based carbonaceous materials. However, due to the intense chemical exothermic reactions that occur during the pre-oxidation of PAN, the PAN fibers are easy burned out if the heat dissipation is not well controlled, especially for the fibers inside the felt, which is a limitation for the development of PAN-ACF felt for chemical protective clothing [19]. In contrast, the liquid-phase pre-oxidation reaction is milder, requiring a shorter treatment time and lower temperature. The pre-oxidized fibers prepared in this manner could exhibit a uniform structure and do not develop a skin-core structure [20]. Therefore, the liquid-phase pre-oxidation method is promising for the fabrication of PAN-ACF felt, although seldom similar works have been reported before. 

In general, an activation process is necessary to regulate the pores in the ACFs. Among various activation strategies, chemical activation based on the KOH is widely used because it can effectively improve the pore structures and enhance the content of the oxygen-containing functional groups of ACFs, which has a positive effect on the adsorption of sulfur compounds [21]. For instance, Li et al. [22] prepared ACFs via a KOH activation method and CO_2_ activation method, respectively, and used them for the adsorption of CS_2_ at room temperature. The result showed that the samples prepared by the KOH activation method exhibited a better adsorption performance for CS_2_. Huang et al. [23] also demonstrated that the ACFs prepared by KOH activation had an amorphous structure, larger specific surface area, and developed micropores. The above results indicate that the KOH activation method can significantly affect the adsorption properties of ACFs, which could also be utilized to improve the adsorption performance of PAN-ACFs for a corresponding chemical warfare agent.

**Figure 1:**
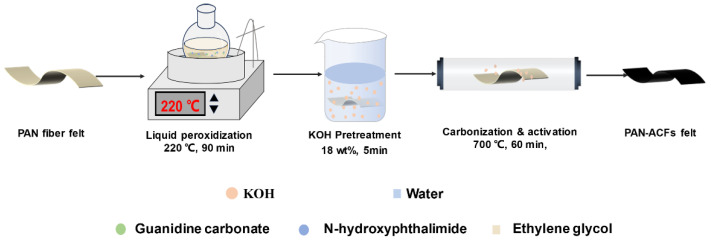
Schematically illustrating the preparation process of the PAN-ACF felt.

In this contribution, an ACF felt with a developed pore structure and chemically functionalized surface was facilely prepared via liquid-phase pre-oxidation as well as one-step carbonization and activation methods. As shown in Figure 1, a needled felt was firstly fabricated using commercial PAN fibers as the raw material, then the obtained PAN fiber felt was pre-oxidized using a liquid-phase pre-oxidation method; finally, the corresponding pre-oxidized PAN fiber felt was carbonized and activated in one step to obtain the ACF felt. The morphology, pore structure, and chemical composition of the as-prepared PAN-ACF felt were characterized and analyzed. The adsorption performance of the corresponding ACF felt for a simulation agent of mustard gas (i.e., dipropyl sulfide) was evaluated, and the results demonstrated that the obtained ACF felt could effectively adsorb the DPS. 

## 2. Materials and Methods

### 2.1. Materials

The PAN fibers (cut into 50–80 mm with an average diameter of 21 μm) were commercially obtained from Toray Co., Ltd. (Tokyo, Japan). Ethylene glycol (98 wt%), guanidine carbonate (99 wt%), N-hydroxy phthalimide (98 wt%), anhydrous ethanol (95 wt%) and dipropyl sulfide (DPS) were purchased from Shanghai McLean Biochemical Technology Co., Ltd. (Shanghai, China), KOH (85 wt%) was purchased from Shanghai Runjie Chemical Reagent Co., Ltd. (Shanghai, China) Deionized water was self-made in the lab.

### 2.2. Preparation of the ACF Felt

PAN fibers with a length of 50~80 mm were used as raw materials to prepare the felts via a common needle-punching method. Then, 100 mL ethylene glycol, 2 g guanidine carbonate and 2 g N-hydroxyphthalimide were mixed and heated to 220 °C in a beaker to obtain the pre-oxidation solution. Subsequently, the as-prepared PAN fiber felt was immersed in the solution at 220 °C for 90 min to carry out the liquid phase pre-oxidation. After that, the pre-oxidized samples were washed with ethanol and deionized water until the detergent was transparent and finally dried at 70 °C for 24 h. The liquid phase pre-oxidation aims to enhance the thermal stability of PAN fibers, ensuring the PAN fiber felt do not burn at elevated temperatures and retain the fibrous structures. To prepare the activated ACF felt, the pre-oxidized fiber felt was immersed in the KOH solution with a concentration of 18 wt% for 5 min; after that, the dried felt was put in a tube furnace and heated to 700 °C with a rate of 10 °C/min and this temperature was maintained for 60 min in the atmosphere of N_2_ to carry out the one-step carbonization and activation. The use of KOH as an activator during activation would result in the generation of a large number of micropores and mesopores in the carbon material. This step is crucial for activated carbon fibers to achieve a high specific surface area [24]. Finally, the obtained ACF felts were taken out the furnace after natural cooling to room temperature and repeatedly washed with deionized water to neutral, and then dried at 70 °C for 24 h.

### 2.3. Characterization

Field emission scanning electron microscopy (FE-SEM, Gemini SEM 300, Oberkochen, Germany) was used to characterize the morphology of the obtained materials at an accelerating voltage of 15 kV. The fiber diameters were obtained based on the obtained SEM images, with 100 fiber diameters measured using Adobe Acrobat 9 Pro software, and the mean and standard deviation were calculated. X-ray diffraction (XRD, Ultima IV, Rigaku, Tokyo, Japan) with a Cu Kα radiation source generated at 40 kV and 40 mA was used to verify the crystal structure and phase compositions of fibers. The surface chemical compositions of the felts were examined by employing X-ray photoelectron spectroscopy (XPS, Ultima IV, Physical Electronics, Chanhassen, MN, USA). The N_2_ adsorption and desorption isotherms of the obtained ACF felt were measured at 77 K using a specific surface area and pore structure analyzer (ASAP-2020M, Micromeritics, Norcross, GA, USA). The specific surface area was calculated by BET method according to the N_2_ adsorption isotherms. The total pore volume was calculated according to the adsorption results of P/P_0_ = 0.995, and the pore size distribution was calculated by the density functional theory (DFT) method.

### 2.4. Adsorption Experiments

The adsorption performance of the obtained ACF felt was evaluated via batch adsorption experiments, using the commonly used sulfur mustard gas simulation DPS as the adsorbates. Typically, 0.02 g ACF felt was added into 100 mL DPS-ethanol solution in a conical flask and shaken at 130 rpm in a constant temperature oscillator. The initial concentration of DPS in the solution was adjusted from 40 to 120 mg/L with the step number of 20 mg/L by changing the ratio of DPS to anhydrous ethanol. In addition, the temperature of the adsorption system was varied from 30 to 60 °C (step number: 10 °C) to study the effect of temperature on the adsorption performance. The residual concentration of DPS in the solution was measured by using an ultraviolet-visible spectrophotometer (TU-1900, Beijing Purkinje General Instrument Co., Ltd., Beijing, China) to analyze the supernatant at a predetermined time interval at 204 nm. The adsorption capacity at equilibrium *q*_e_ (mg/g) was calculated using the following equation:

(1) $$q_{e}=\left(\frac{C_{0}-C_{e}}{W}\right)\times V$$

where *C*_0_ and *C*_*e*_ (mg/L) are the initial and equilibrium concentrations of the DPS solution, *W* (g) is the mass of the adsorbent, and *V* (L) is the volume of the solution.

### 2.5. Adsorption Kinetics Analysis

To investigate the adsorption mechanism of DPS on the ACF felt, the pseudo-first-order and pseudo-second-order adsorption kinetic models as well as the intraparticle diffusion model were used to analyze the adsorption process. The three kinetic model equations are as follows [25,26].

The pseudo-first-order adsorption kinetic model is represented by the following linear form:

(2) $$\ln\left(q_{e}-q_{t}\right)=\ln\left(q_{e}\right)-k_{1}t\;$$

where *k*_1_ (min^−1^) is the pseudo-first-order adsorption rate constant, and *q*_*e*_ (mg/g) and *q*_t_ (mg/g) are the adsorption capacity at equilibrium and at time *t*, respectively. According to the slope and intercept of the fitted linear graph, *k*_1_ and *q*_*e*_ can be calculated, respectively. 

The pseudo-second-order adsorption kinetic model is represented by the following linear form:

(3) $$\frac{t}{q_{t}}=\frac{1}{k_{2}q_{e}^{2}}+\frac{t}{q_{e}}$$

where *k*_2_ (g/(mg∙min)) is the pseudo-second-order adsorption rate constant.

The intraparticle diffusion model is described by the following equation:

(4) $$q_{t}=k_{\mathrm{int}}t^{1/2}+C\;$$

where *k*_int_ (mg/(g∙min^1/2^)) is the rate constant of intraparticle diffusion; *C* is the intercept of the fitted linear plot of *q*_t_ against *t*^1/2^, which is proportional to the thickness of the boundary layer, and can be used to judge whether intraparticle diffusion is the only rate-controlling step or not.

### 2.6. Adsorption Equilibrium Isotherm

The equilibrium adsorption data of DPS on the ACF felt were fitted by the two most commonly used isotherm models, namely, the Langmuir and Freundlich isotherm models.

The Langmuir isotherm assumes a surface with homogeneous adsorption sites, equivalent adsorption energies, and no interaction between adsorbed species [27]. It is expressed as

(5) $$\frac{C_{e}}{q_{e}}=\frac{C_{e}}{q_{m}}+\frac{1}{K_{L}q_{m}}$$

where *q*_m_ (mg/g) and *K*_L_ (L/mg) are the Langmuir constants related to the adsorption capacity and adsorption rate, respectively, with *q*_m_ representing the maximum monolayer coverage of adsorbent with adsorbate and *K_L_* representing the adsorption energy. *q*_m_ and *k*_L_ can be calculated according to the slope and intercept of the fitted linear plot.

The essential characteristics of the Langmuir isotherm can be expressed by a dimensionless equilibrium parameter *R_L_* [28], which is defined by

(6) $$R_{L}=\frac{1}{1+K_{L}C_{0}}$$

where *C*_0m_ (mg/L) is the highest initial DPS concentration. The value of *R*_*L*_ indicates the type of the isotherm to be either unfavorable (*R_L_* > 1), linear (*R_L_* = 1), favorable (0 < *R_L_*< 1), or irreversible (*R_L_* = 0).

The Freundlich isotherm model is an empirical equation based on an exponential distribution of adsorption sites and energies and assumes that the adsorption of the adsorbate on the multiphase surface is multi-molecular layer adsorption [29]. It is expressed as

(7) $$\ln(q_{e})=\ln(K_{F})+\frac{1}{n}\ln(C_{e})$$

where *K*_F_ and 1/n are Freundlich constants related to adsorption capacity and adsorption intensity, respectively, with *K*_F_ representing the relative adsorption capacity of the adsorbent and 1/n representing the degree of dependence of adsorption on the *C*_e_. *K*_F_ and 1/n can be calculated according to the intercept and slope of the linear fitting plot, respectively.

### 2.7. Adsorption Thermodynamic

The thermodynamic study of the adsorption of DPS by the ACF felt was carried out because the thermodynamic parameters are important to assess the adsorption spontaneity and solid–liquid interfacial stability. The thermodynamic parameters include Gibbs free energy (Δ*G*), enthalpy (Δ*H*), the thermodynamic equilibrium constant (*K_G_*), and entropy (Δ*S*); the equations involved in the calculations are shown below [30]:

(8) $$K_{G}=\frac{C_{e}}{q_{e}}$$

(9) $$\ln K_{G}=\frac{\Delta S}{R}-\frac{\Delta H}{RT}$$

(10) $$\Delta G=-RT\ln K_{G}$$

where *R* is the universal gas constant, with a value of 8.314 J/(mol·K); Δ*T* is the thermodynamic temperature; Δ*H* is the enthalpy of light adsorption; and Δ*S* is the macroscopic intrinsic complexity of the table. Δ*H* and Δ*S* can be determined from the slope and intercept of a linear fit plot of ln *K_G_* to 1/*T,* respectively.

## 3. Results and Discussions

### 3.1. Morphologies of the PAN Fiber Felt and ACF Felt

**Figure 2:**
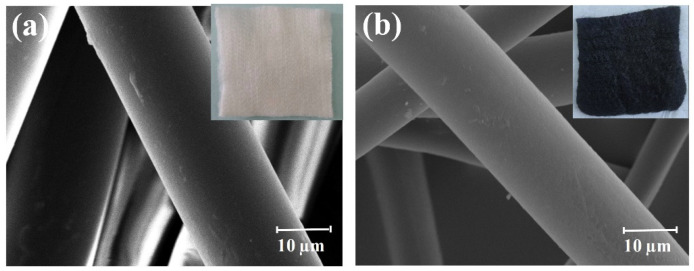
SEM images of (**a**) PAN fiber felt and (**b**) ACF felt with corresponding macro photographs inserted.

Figure 2 gives the SEM images and digital photos of the PAN fiber felt and corresponding ACF felt. It can be seen that the pristine PAN fiber felt was white and exhibited a typical fibrous structure (Figure 2a), while the obtained ACF felt was black and kept an intact macro structure (Figure 2b). Moreover, the SEM image of the ACF felt indicates that the fibrous structure of the pristine PAN fiber felt was well inherited. The average diameter of the ACFs was about 21 μm, which was 14.3% lower than that of the PAN fiber, which was mainly the result of the elimination of non-carbon elements and ablation of the carbon matrix during the one-step carbonization and activation [31]. 

### 3.2. Evolution of the Microstructures of Fibers

**Figure 3:**
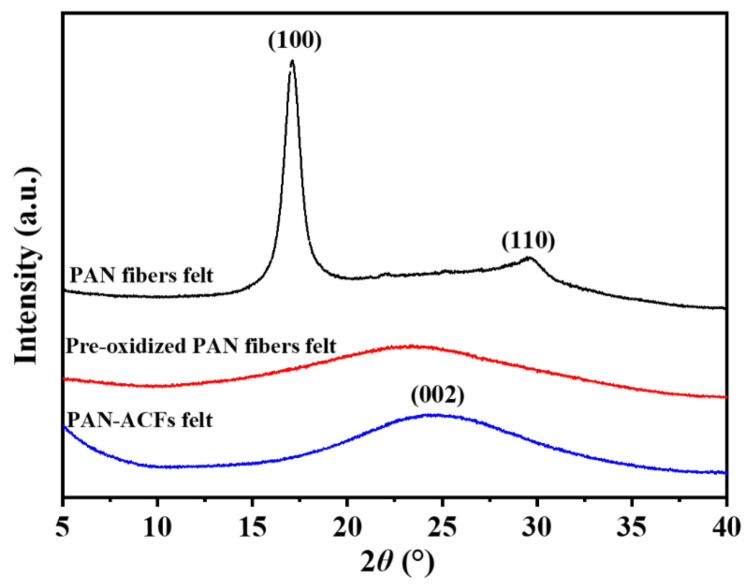
XRD patterns of the PAN fiber felt, pre-oxidized fiber felt and corresponding ACF felt.

Figure 3 shows the X-ray diffraction patterns of the pristine PAN fiber felt, pre-oxidized PAN, and corresponding ACF felt. The XRD spectrum of the pristine PAN fiber felt shows strong characteristic diffraction peaks at 2*θ* = 17.12° and 2*θ* = 29.58°, corresponding to the (100) and (110) planes of the crystal structure of PAN, respectively. According to the Bragg equation 2*d*sin*θ* = *nλ* [32] (where *n* = 1, *λ* = 0.1541 nm, *d* is the lattice spacing; *θ* is half of the diffraction angle), the lattice spacing was calculated to be *d*_16.64_ = 0.518 nm and *d*_29.08_ = 0.302 nm, respectively, which is consistent with the results of the previous study [33]. After the pre-oxidization, the characteristic diffraction peaks of PAN disappeared and a wide peak around at 2*θ* = 22.6° could be observed, which means that the original crystalline structure of the PAN fiber had been destroyed due to the cyclization reaction of the PAN molecular chain [34]. From the XRD spectrum of ACF felt, it can be seen that a new wide diffraction peak appears at 2*θ* = ~24.88°, which belongs to the characteristic peak of amorphous carbon [35]. The calculated lattice spacing (d_24.88_ = 0.358 nm) was smaller than that of the pre-oxidized fibers (d_22.6_ = 0.393 nm). 

### 3.3. Surface Chemical Properties 

**Figure 4:**
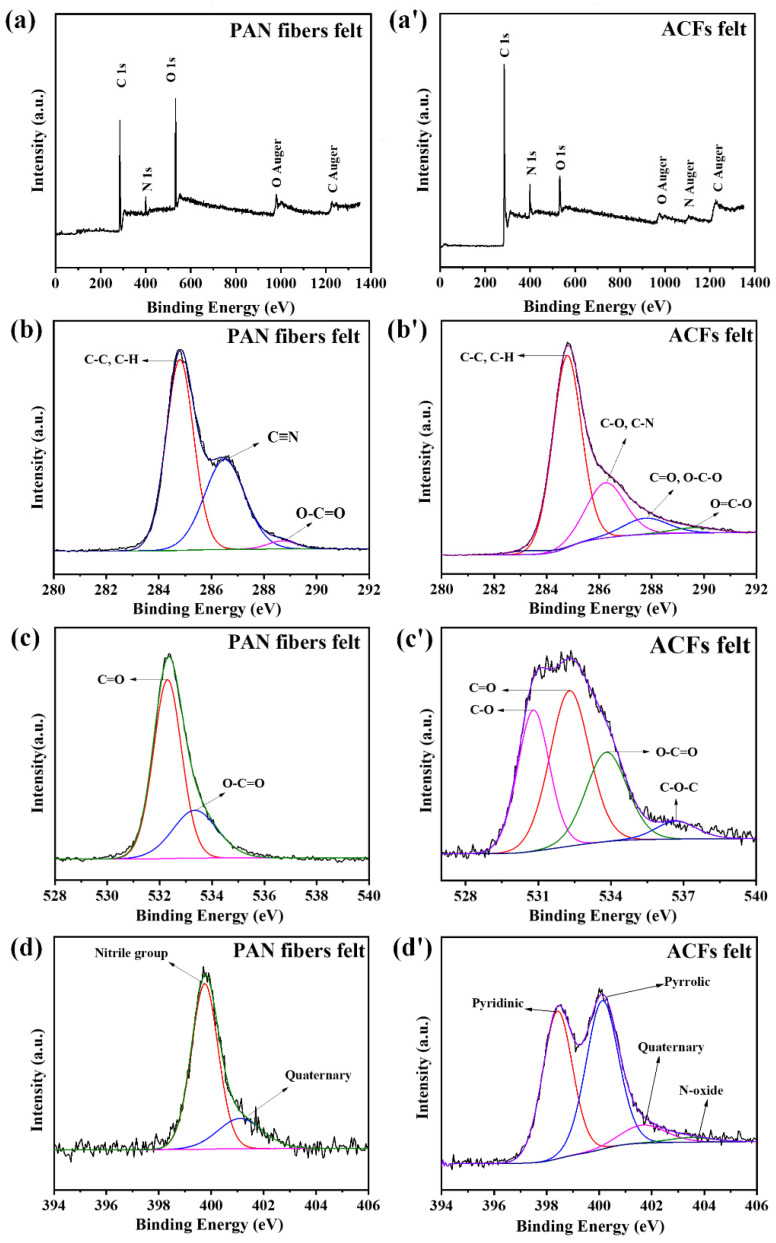
XPS spectra of PAN fibers and related ACFs: (**a**,**a**′) survey spectra; (**b**,**b**′) C1s spectra; (**c**,**c**′) O1s spectra; and (**d**,**d**′) N1s spectra.

The chemical structure of the PAN fiber felt and ACF felt were studied using XPS analysis. It can be seen from Figure 4a,a′ that the XPS survey spectra of both the PAN fibers and ACFs felts showed main peaks of the C, N, and O elements. The relatively high oxygen content in PAN fiber felt may be attributed to the presence of copolymer-containing oxygen elements. Meanwhile, it was found that the relative intensity of O was significantly reduced in the curve of ACF felt, which may be due to the elimination of O during the carbonization process of PAN fibers. The peaks around at 976, 1100, and 1226 eV were associated with the Auger peaks of elements O, N, and C, respectively [36]. Generally, in an Auger process, the energy released occurs when an electron transitions from a higher energy level to a deeper level. This transition could lead to the emission of an energetic electron, whose energy was determined by the specific electron levels involved [37]. For the PAN fiber felt, deconvolution of the C 1s spectrum could be resolved in in C≡N, O-C=O, and carbon in C-C or C-H, while for the ACF felt, four signals at 284.8, 286.3, 287.8, and 289.7 eV could be obtained, which were related to C-H/C-C, C-O/C-N, C=O/O-C-O, and O=C-O, respectively [38,39]. The C-N was derived from the broken C≡N bond of the PAN [40]. Figure 4c,c′ shows the peak fitting diagram of the O 1s spectrum. It is apparent that the split O 1s curve of the PAN fiber felt displayed two distinct signals at 532.2 eV and 533.4 eV, which could be attributed to the presence of oxygen atoms in hydroxyls and ethers, as well as carboxylic acids, respectively. The O1s spectrum of the corresponding ACF felt could be resolved in four peaks related to the carbonyl oxygen atoms in the carbonyl, quinone, ether, carboxyl and lactone groups, respectively [41,42]. As the ACF felt were derived from the PAN precursor, a deeper examination at N 1s peak was necessary to understand the characteristics of the materials. Figure 4d shows the peak fitting diagram of the N 1s spectrum of the PAN fiber felt, from which two peaks around at 399.8 and 401.2 eV could be observed, which could be attributed to the nitrile group and quaternary-N [43]. For the N 1s spectrum of the ACF felt (Figure 4d′), three signals around 398.4, 400.1, and 401.7 eV, and 403.4 eV could be obtained, which were related to the pyridinic-N, pyrrolic-N, quaternary-N, and N-oxide, respectively [44,45,46,47,48]. The diverse status of N in the PAN fibers and corresponding PAN-ACFs may be caused by the different chemical environment and configuration [49,50]. Similar nitrogen-containing mixtures showing content changes after heat treatment can also be found in other studies [51]. 

### 3.4. Specific Surface Area and Pore Structure Analysis

**Figure 5:**
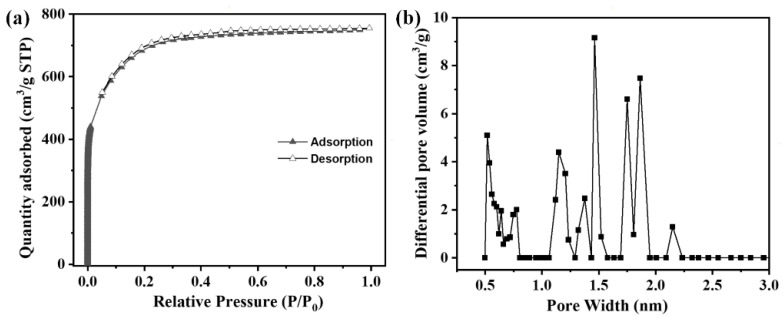
(**a**) N_2_ adsorption–desorption isotherms and (**b**) pore size distribution curve of the ACF felt.

Figure 5 shows the N_2_ adsorption–desorption isotherms and pore size distribution of the related ACF felt. It can be seen that the adsorption–desorption isotherm could be assigned to type I with a high N_2_ adsorption capacity in the low-pressure region, and an obvious platform in the middle region. This phenomenon indicates that the pores in ACFs were rapidly filled below the relative pressure of about 0.2, and kept almost constant at a higher relative pressure, indicating that the ACFs were highly micro-porous. In addition, a weak adsorption hysteresis appeared at a higher relative pressure, indicating the presence of a small amount of mesopores. When the relative pressure reached 0.95, the N_2_ adsorption capacity increased slightly, which was caused by the capillary condensation of macro-pores [52] and was consistent with the pore size distribution curve shown in Figure 5b. The corresponding BET specific surface area of the ACF felt was 2219.48 m^2^/g with a total pore volume of 1.168 cm^3^/g. The average pore size of the ACFs was 2.1 nm, and the percentage of the micropore and mesopore was 98.29% and 1.71%, respectively, suggesting that the porous structures of the obtained ACF felt were dominated by micropores. 

The developed pore structure of the obtained ACF felt was mainly contributed to by the chemical activation effect of KOH on the as-formed carbon matrix of fibers during the one-step carbonization and activation. In general, when the temperature was below 450 °C, complete water loss occurred in the PAN fibers. In addition, the KOH adsorbed by the PAN fiber felt would melt and selectively react with the volatile substances in the fibers. With the carbonization temperature increased to the range of 450 and 650 °C, a great amount of gases, including hydrogen, ammonia, and so on, were generated due to the direct reaction between KOH and pyrolytic carbon, resulting the formation of numerous micropores. In addition, the removal of volatile/isomers could also bring a notable increase in the porosity. At higher temperatures (>700 °C), predominant pore enlargement happened when the previously generated metal compounds or monomers of potassium boiled, further contributing to the pore enlargement process [53].

### 3.5. Adsorption Properties of the ACF Felt towards DPS

**Figure 6:**
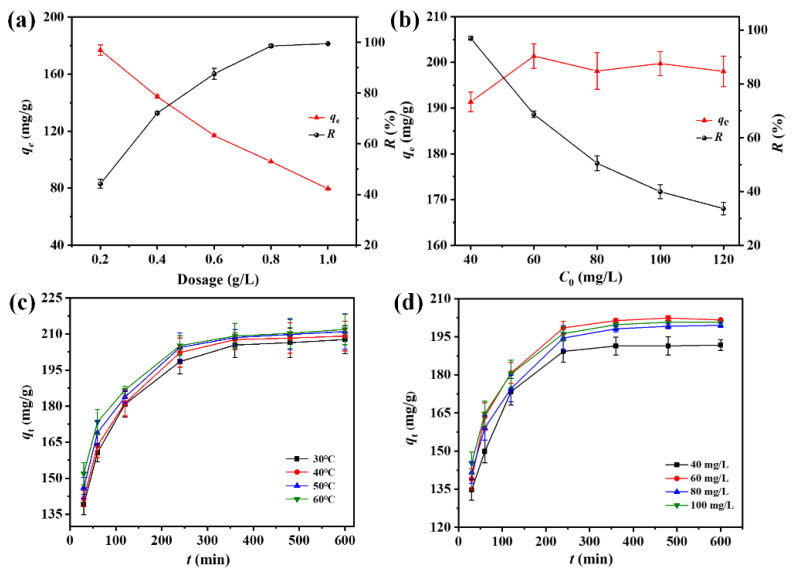
(**a**) Curves demonstrating the *q_e_* and *R* with different dosages of ACFs (experimental condition: *T* = 25 °C, *C*_0_ = 80 mg/L); (**b**) curves of the *q_e_* and *R* with different initial DPS concentrations (experimental condition: *T* = 25 °C, dosage = 0.2 g/L); (**c**) kinetic adsorption curves at different temperatures (experimental condition: *C*_0_ = 80 mg/L, dosage = 0.2 g/L); and (**d**) kinetic adsorption curves under different initial concentrations of DPS (experimental condition: *T* = 25 °C, dosage = 0.2 g/L).

Figure 6a demonstrates the effects of the dosage on the adsorption properties of ACF felt towards DPS. It was found that with an increase in the dosage, the adsorption capacities ($q_{e}$) gradually decreased, which could be attributed to the fact that the number of DPS molecules that could be adsorbed by the ACFs in the solution was limited; thus, excessive dosage would result in unused binding sites of the ACFs [54,55]. On the contrary, the corresponding removal efficiency (*R*) increased obviously and achieved almost 100% when the dosage was 1 g/L. This could be mainly attributed to the raise in free active sites and availability of a larger surface area when increasing the amount of absorbent [56,57]. Figure 6b depicts the evolution of $q_{e}$ and *R* with the change in the initial DPS concentration (*C*_0_). It can be found that, with the increase in *C*_0_, the $q_{e}$ increased at first and then gradually reached a dynamic equilibrium, while the *R* kept decreasing. This was due to the fact that within a certain range, the higher the DPS concentration, the greater the adsorption drive provided to the ACFs; thus, a higher $q_{e}$ could be obtained [58]. However, when the concentration exceeded this range, the adsorption sites of the ACFs were saturated. Even if the *C*_0_ continued to increase, no more DPS molecules could be adsorbed; thus, a dynamic adsorption equilibrium could be obtained. A similar phenomenon can also be found in a previous study [59]. Figure S1 demonstrates the effect of the pH value on the adsorption properties of the ACF felt for DPS. It was found that the adsorption capacity increased with the increase in the pH values, indicating that the initial pH of the solution played a crucial role in the adsorption process [60]. The enhanced adsorption capacity of the ACFs for the DPS in a solution with a higher pH value may be attributed to the point of the zero charge potential (pH_pzc_) of ACFs, which was found to be between 2 and 3 [61]. When pH > pH_pzc_, the ACF surface inclined to adsorb anions [62]; therefore, an enhanced electrostatic attraction force between the surface of ACFs and DPS molecules would be obtained. 

The kinetic adsorption curves at different temperatures, shown in Figure 6c, demonstrate that during the whole adsorption process, the instant adsorption capacity ($q_{t}$) increased greatly at the initial stage of adsorption and then tended to be balanced, which meant that the adsorption had reached an equilibrium state. Meanwhile, the adsorption capacity of DPS on ACFs at the same adsorption time gradually increased with the increase in temperature, indicating that the higher temperature could improve the adsorption capacity [63]. Figure 6d shows the kinetic adsorption curves with different initial concentrations of DPS solution. It can be observed that the kinetic adsorption behavior of DPS on ACFs at different times were similar. But the $q_{t}$ with a *C*_0_ of 60 mg/L was much higher than that of 40 mg/L, and no obvious changes of $q_{t}$ could be observed when further increasing the value of *C*_0_, which was well in agreement with the results shown in Figure 6b.

### 3.6. Adsorption Kinetic Analysis

**Figure 7:**
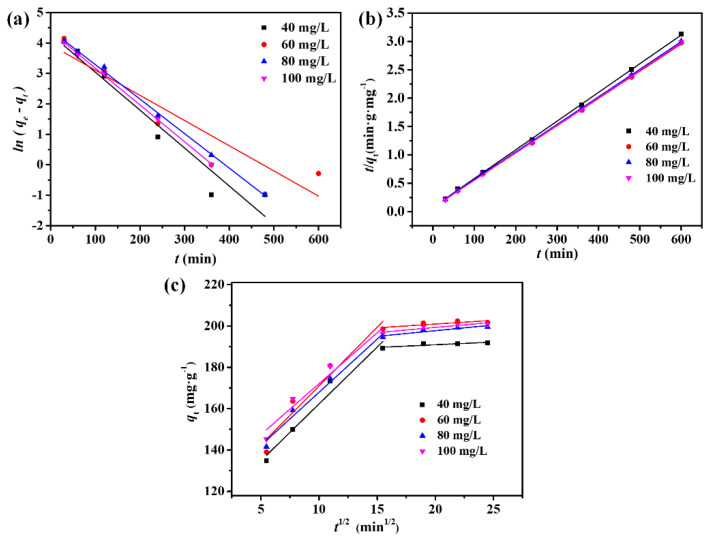
Fitting plots of the adsorption kinetics of DPS on the ACFs: (**a**) pseudo-first-order kinetic model; (**b**) pseudo-second-order kinetic model; and (**c**) intraparticle diffusion model.

To obtain a comprehensive understanding of the adsorption process, the adsorption kinetics of DPS on ACFs under various initial DPS concentrations were further analyzed using the pseudo-first-order and pseudo-second-order adsorption kinetic models, as well as the intraparticle diffusion model, as described by Equations (2)–(4). The fitted curves are presented in Figure 7, and the corresponding kinetic parameters are listed in Table 1 and Table 2. It can be seen from Figure 7a,b that the adsorption process of DPS on ACFs could be well-fitted with the pseudo-second-order kinetic model, with higher values of *R*^2^ than those of the pseudo-first-order kinetic model (Table 1). Moreover, the calculated *q*_e_ values (*q*_e cal_) obtained from the pseudo-second-order equation also appeared to agree well with the experimental *q*_e_ data (*q*_e exp_), indicating that the pseudo-second-order kinetic model could better describe the whole adsorption process. According to the fitting results of the pseudo-second-order adsorption kinetic model, the adsorption of DPS on the obtained ACF felt was dominated by chemical adsorption.

Figure 7c demonstrates that all the fitting curves exhibited a two-stage linear trend, confirming the presence of intraparticle diffusion. As shown in Table 2, the K_int_ of the first stage was much higher than that of the second stage, and the C value of the second stage was significantly increased, indicating that the boundary layer had a notable effect on the adsorption process of DPS on the ACFs. The first stage could be attributed to the boundary layer diffusion of DPS molecules, that is, external diffusion, while the second stage represented the slow adsorption process, where intraparticle diffusion became the main factor affecting the adsorption rate. In other words, the adsorption of DPS molecular on the ACFs was thought to have occurred through external adsorption at first, until the surface adsorption sites were fully occupied (first stage). Subsequently, DPS molecules diffused into the internal pores of the ACFs for further adsorption (second stage). It can be observed from Table 2 that the first and second stages did not pass through the origin (C ≠ 0), indicating that intraparticle diffusion was not the only rate-controlling step for the adsorption of DPS on the ACFs, and the thickness of the boundary layer also had a certain effect on the adsorption rate.

### 3.7. Adsorption Equilibrium Analysis

**Figure 8:**
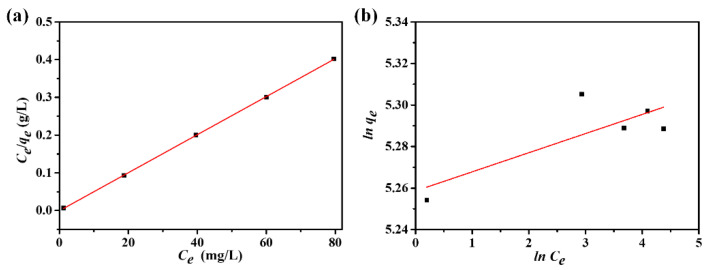
Adsorption equilibrium analysis of DPS on the ACF felt: (**a**) Langmuir isotherm model; (**b**) Freundlich isotherm model.

The adsorption isotherms of DPS on the ACFs were further analyzed according to the Langmuir and Freundlich isotherm models using Equations (5) and (7). The fitting plots are shown in Figure 8, and the corresponding fitting parameters are listed in Table 3. It can be observed that the linear correlation coefficient *R*^2^ of the Langmuir isotherm model was 0.9999, which was much higher than that of the Freundlich isotherm model (*R*^2^ = 0.5203). Moreover, the calculated *q*_m_ value (198.41 mg/g) was very close to the experimental *q*_e_ values (*q*_e exp_), suggesting that the adsorption process of DPS on the ACFs could be well described by the Langmuir isotherm model. The conformation of the experimental data into the Langmuir isotherm model indicates that monolayer adsorption of DPS had taken place on the surface of pores in the ACFs. According to Equation (6), the dimensionless parameter, the *R_L_* value, was obtained. The *R_L_* value of the adsorption process was calculated to be 0.0129, which further confirmed that the adsorption of DPS on the ACFs was favorable under the experimental conditions used in this study.

### 3.8. Adsorption Thermodynamics Analysis

**Figure 9:**
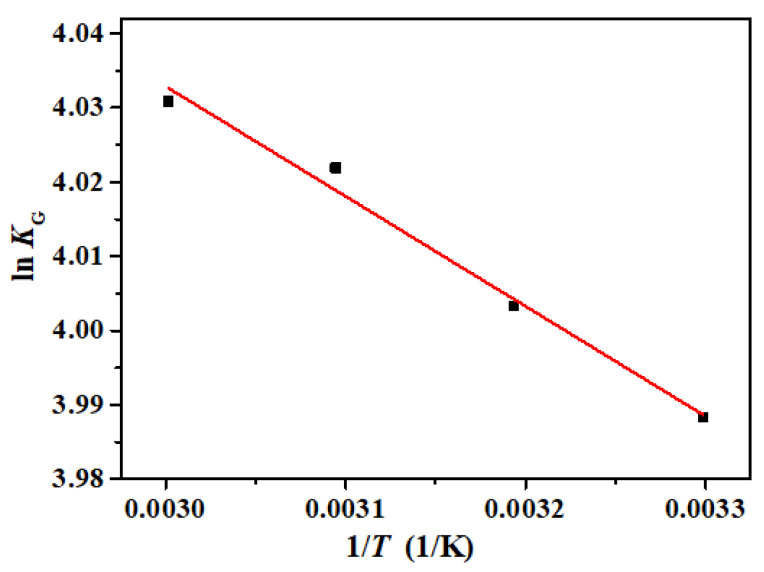
The linear fitting curve of lnK_G_ to *T*^−1^ by the Van’t Hoff equation.

Adsorption thermodynamics can be used to analyze the spontaneity and feasibility of adsorption and to speculate the adsorption mechanism. The results of the linear fitting of lnK_G_ to *T*^−1^ by the Van’t Hoff equation are shown in Figure 9. The corresponding thermodynamic fitting parameters are listed in Table 4. It can be seen that the Δ*H* is 1.23 kJ/mol, which is positive, indicating that the adsorption process of DPS on the ACFs was an endothermic reaction. The Δ*S* was 37.22 J/(mol·K), which was also positive, indicating that the concentration of adsorbate at the solid/liquid interface increases during the adsorption process. The positive values of Δ*H* and Δ*S* further verify that the adsorption is a chemical adsorption process [64]. The negative value of Δ*G* indicates the spontaneity and feasibility of the adsorption process. With the increase in temperature, the value of Δ*G* decreased from −10.05 kJ/mol to −11.16 kJ/mol, indicating that the adsorption was more favorable at higher temperatures.

### 3.9. Adsorption Mechanism of DPS towards ACFs

**Figure 10:**
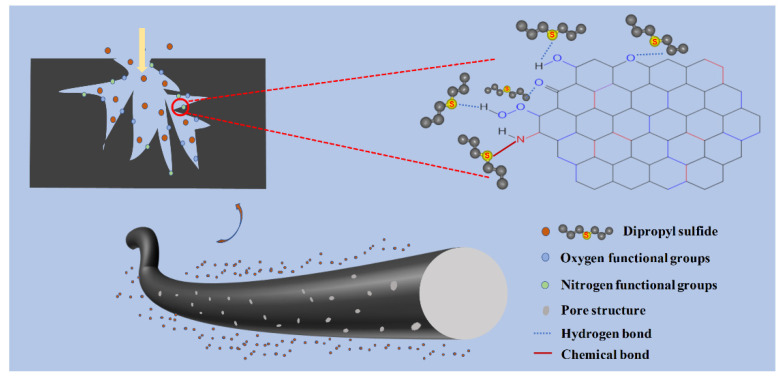
Schematic illustration of the plausible adsorption mechanism of ACFs towards DPS.

Figure 10 schematically shows a plausible mechanism for the DPS adsorption on the ACF felt. The abovementioned results have demonstrated that the as-prepared ACF felt possessed a high specific surface area and developed pore structure, which could provide sufficient channels and adsorption sites for the transmission and adsorption of DPS [65]. The adsorption kinetics and adsorption equilibrium analysis showed that the adsorption of DPS on the ACF felt was dominated by the chemical adsorption process, which may be caused by the abundant oxygen-containing functional groups and nitrogen-containing functional groups on the fiber surface [66]. These oxygen-containing functional groups, such as carboxyl (COO-), hydroxyl (-OH), carbonyl (C=O), a carbon-oxygen single bond (C-O) and an ether bond (C-O-C), could increase the surface polarity of ACFs and easily form hydrogen bonds with DPS molecules. Additionally, the presence of nitrogen-containing functional groups could also provide more chemical binding sites for the chemical adsorption of DPS molecules, thereby enhancing the adsorption effect of DPS molecules on ACF [67]. Moreover, the van der Waals interaction force between the DPS molecules and the ACFs may also have existed due to the high surface area and developed pores, which could form the physical adsorption effect; thus, the adsorption process of ACF felt towards DPS should be a synergistic effect of physical adsorption and chemical adsorption. 

## 4. Conclusions

In summary, an ACF felt with developed pore structures was prepared via liquid-phase pre-oxidation, as well as one-step carbonization and chemical activation. The obtained ACF felt possessed a large specific surface area (2219.48 m^2^/g) and developed pore structure (pore volume: 1.168 cm^3^/g; average pore size: 2.1 nm; micro-pore rate: 98.29%), as well as rich oxygen/nitrogen-containing functional groups on the surface of the ACFs. As a result, the ACF felt demonstrated a good adsorption performance towards DPS. The maximum adsorption capacity was up to 202.38 mg/g. The adsorption kinetics studies showed that the pseudo-second-order adsorption model could well describe the adsorption process of DPS on the ACF felt and the adsorption equilibrium data conformed to the Langmuir isotherm model. The intraparticle diffusion analysis showed that it was a two-stage adsorption process, and the intraparticle diffusion was the rate-controlling step. Considering the high surface area, developed pores, and various functional groups on the surface of the ACFs, the adsorption of DPS on the ACF felt may have been a synergistic effect of physical adsorption and chemical adsorption, but the chemical adsorption dominated. The physical adsorption was due to the van der Waals interaction force between the DPS molecules and the ACFs, while the chemical adsorption effect mainly resulted from the hydrogen bonds or chemical bonds formed between the DPS molecules and the polar groups on the surface of the ACFs. 

## Figures and Tables

**Table 1:** Fitting parameters of the pseudo-first-order and pseudo-second-order adsorption kinetic models.

*C*_0_	*q*_e exp_ (mg/g)	Pseudo-First-Order Kinetic Model			Pseudo-Second-Order Kinetic Model		
		*k*_1_ (1/min)	*q*_e cal_ (mg/g)	*R* ^2^	*k*_2_ (g·mg^−1^·min^−1^)	*q*_e cal_ (mg/g)	*R* ^2^
40	191.75	2.87 × 10^−2^	193.01	0.9359	3.37 × 10^−4^	197.63	0.9997
60	202.38	1.90 × 10^−2^	86.19	0.8557	3.23 × 10^−4^	207.90	0.9998
80	199.50	2.61 × 10^−2^	264.05	0.9978	3.00 × 10^−4^	205.76	0.9998
100	200.75	2.78 × 10^−2^	240.71	0.9983	3.47 × 10^−4^	206.17	0.9999

**Table 2:** Fitting parameters of the intraparticle diffusion model.

*C*_0_	The First Stage			The Second Stage		
	*K*_int_ [mg/(g∙min^1/2^)]	*C*	*R* ^2^	*K*_int_ [mg/(g∙min^1/2^)]	*C*	*R* ^2^
40	5.50	107.30	0.9852	0.26	185.71	0.8798
60	5.68	114.12	0.9733	0.36	193.63	0.8274
80	5.14	116.74	0.9924	0.55	186.72	0.9308
100	4.92	122.86	0.9801	0.50	189.27	0.9066

**Table 3:** Fitting parameters of Langmuir isotherm and Freundlich isotherm.

Langmuir Isotherm			Freundlich Isotherm		
*q*_m_ (mg/g)	*K*_L_ (L/mg)	*R* ^2^	*K*_F_ (mg/g)	1/*n*	*R* ^2^
198.41	0.957	0.9999	252	9.21 × 10^−3^	0.5203

**Table 4:** Adsorption thermodynamic fitting parameters.

Δ*S* (J∙mol^−1^∙K^−1^)	Δ*H* (kJ/mol)	Δ*G* (kJ/mol)			
		30 °C	40 °C	50 °C	60 °C
37.22	1.23	−10.05	−10.42	−10.81	−11.16

## Data Availability

Data are contained within the article and supplementary materials.

## Supplementary Materials

The following supporting information can be downloaded at: https://www.mdpi.com/article/10.3390/polym16020252/s1, Figure S1: Effect of initial pH on adsorption performance of ACF felt towards DPS.
