# Supplementary material for: Facile Preparation of Polyacrylonitrile-Based Activated Carbon Fiber Felts for Effective Adsorption of Dipropyl Sulfide
Source: Polymers (Basel). 2024 Jan 16;16(2):252. doi: 10.3390/polym16020252 (PMC10820270; doi:10.3390/polym16020252)
Supplement: Supplementary file 1 [file polymers-16-00252-s001.zip › polymers-2691839-supplementary.pdf]

Supporting information

Facile preparation of polyacrylonitrile-based activated carbon fiber felts for effective adsorption of dipropyl sulfide

Tianhao Zhang [1†], Yafang He[1†], Shiqi Hu[1], Jianlong Ge [1,*], Tianye Chen [2,*], Haoru Shan [1], Tao Ji [1], Decheng Yu [2] and Qixia Liu [1,*]

[1] National & Local Joint Engineering Research Center of Technical Fiber Composites for Safety and Protection, School of Textile and clothing, Nantong University, Nantong, China
[2] Jiangsu Sutong Carbon Fiber Co., Ltd, Nantong, Jiangsu, China
† The author contributed equally to this work (T. Z. and Y. H.)
* Correspondence: gejianlong@ntu.edu.cn (J. G.); cty@stacf.com (T. C.); lqx@ntu.edu.cn (Q. L.)

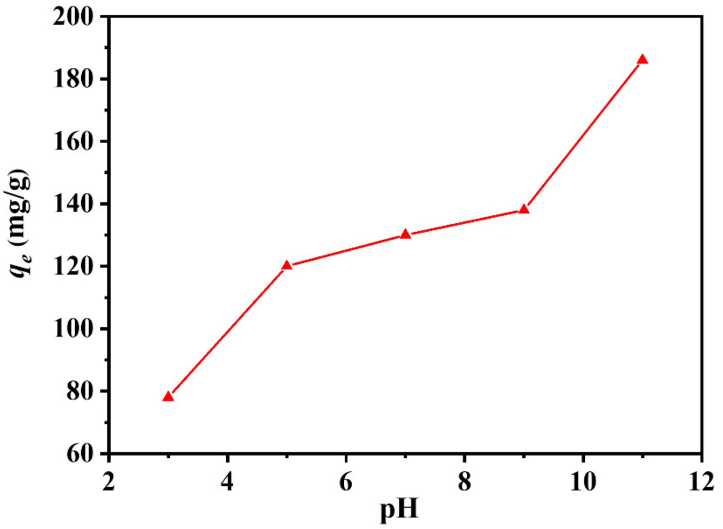

Figure S1. Effect of initial pH on adsorption performance of ACFs felt towards DPS.
